# Health Impacts of Climate Change in the Solomon Islands: An Assessment and Adaptation Action Plan

**DOI:** 10.5539/gjhs.v6n5p261

**Published:** 2014-06-24

**Authors:** Jeffery T Spickett, Dianne Katscherian

**Affiliations:** 1 Faculty of Health Sciences, School of Public Health WHO Collaborating Centre for Environmental Health Impact assessment, Curtin University, Perth, Western Australia, Australia

**Keywords:** climate change, health impact assessment, adaptation, the Solomon Islands

## Abstract

The Pacific island countries are particularly vulnerable to the environmental changes wrought by global climate change such as sea level rise, more frequent and intense extreme weather events and increasing temperatures. The potential biophysical changes likely to affect these countries have been identified and it is important that consideration be given to the implications of these changes on the health of their citizens.

The potential health impacts of climatic changes on the population of the Solomon Islands were assessed through the use of a Health Impact Assessment framework. The process used a collaborative and consultative approach with local experts to identify the impacts to health that could arise from local environmental changes, considered the risks associated with these and proposed appropriate potential adaptive responses. Participants included knowledgeable representatives from the biophysical, socio-economic, infrastructure, environmental diseases and food sectors.

The risk assessments considered both the likelihood and consequences of the health impacts occurring using a qualitative process. To mitigate the adverse effects of the health impacts, an extensive range of potential adaptation strategies were developed. The overall process provided an approach that could be used for further assessments as well as an extensive range of responses which could be used by sectors and to assist future decision making associated with the Solomon Islands’ responses to climate change.

## 1. Introduction

The Intergovernmental Panel on Climate Change (IPCC) 4^th^ Assessment report has stated that, globally, increased mortality and morbidity as a consequence of climate change has commenced and will continue (IPCC, 2007). It is therefore imperative to attempt to identify and evaluate the likely changes to the environment and the direct and indirect impacts on the health of the community. To respond to climate change adaptation strategies need to be developed and applied to ensure that any ensuing adverse health impacts are minimised (Cambell-Lendrum et al., 2006).

In order to plan effective adaptation strategies the ways in which the environment and human health interact with climatic variability must be better understood (WHO, 2003a). This will then allow predictions of likely health impacts to be made although some uncertainty will be inevitable. The strategies that are developed will need to be tailored to specific locations because climate change effects will not be uniform around the globe. It should also be recognised that health effects can be either adverse or beneficial. Those that are beneficial must be maximised while strategies that minimise the adverse effects must be available for implementation.

Some stepwise pathways of health impacts associated with climate change were initially proposed by a WHO Working Group in 1990 and it was noted that these could result from direct or indirect exposures (WHO, 1990). The determinants of health affected by climate changes are those associated with social and economic environments, the biophysical environment and each person’s individual characteristics and behaviour. Climate-related factors that can lead to direct exposures include fires, floods and heatwaves while climate changes that affect environmental factors such as air, water, vectors of disease and food production and quality can result in indirect exposures as can social parameters such as changing economic variables and distributions of population (IPCC, 2007). Opportunities for the application of adaptation strategies can be identified where points of vulnerability occur at any of the steps in health impact pathways. Vulnerability to climate change is considered to be the degree to which a system is susceptible to or unable to cope with the adverse effects that occur (IPCC, 2001) and is a function of exposure to climatic factors, sensitivity to change and adaptive capacity. The first factor depends on location and activities while sensitivity is related to the ways in which the individual, community or system responds to the change. Adaptive capacity refers to the capacity of institutions, systems and individuals to adjust to climate changes as well as ability to take advantage of opportunities and to cope with the consequences.

The mitigation of health impacts is not always the responsibility of the health sector so a collaborative approach is essential. Involvement of a range of sectors must occur in the development and implementation of the necessary strategies to enable optimal responses to the various risk factors that determine vulnerability. In particular it is important to ensure that a comprehensive assessment of vulnerability occurs since the main benefits of adaptation measures take place when critical points in the pathway and/or vulnerable sectors of the population are the main focus of these measures.

### 1.1 Climate Change and Health in the Pacific Island Nations

At the 2009 biennial meeting of the Ministers for Health of the Pacific island countries in Madang, Papua New Guinea discussions included the impacts on health which could occur as a consequence of climate change. In 2008 the WHO had produced a pivotal document entitled Regional Framework for Action (on Climate Change and Health). This Framework set out key guidelines and core responsibilities for the health sector to protect communities from the health impacts of climate change in the Asia Pacific region (WHO, 2008). Following the 2009 meeting the Ministers realised that the Pacific Island nations would be especially vulnerable to the impacts of climate change, identified some common climate-sensitive health risks and were a high priority, throughout the region (WHO, 2009).

The Ministers were prepared to commit to action on climate change and health. They recommended that each Pacific island country plan and implement activities that would increase knowledge and understandings of the health impacts and vulnerability of their communities to climate change and develop adaptation strategies and action plans in collaboration with key sectors and other stakeholders. These recommendations were consistent with those proposed in the World Health Organization’s (WHO) “Regional Framework for action to protect human health from effects of climate change in the Asia Pacific region” (WHO, 2008).

Within the climate change arena in the Solomon Islands the Ministry of Environment, Conservation and Meteorology (MECM) had undertaken a significant amount of activity and adaptation planning. One of the key references for such activities is the Solomon Islands National Adaptation Programme of Action (NAPA, 2008), which ranks health alongside agriculture and food security, water and human settlements as the priority sectors for climate change adaptation. The Solomon Islands 2^nd^ National Communication to the United Nations Framework Convention on Climate Change (UNFCCC) and the National Climate Change Policy documents provide the foundation for adaptation and mitigation strategies to protect the Solomon Islands communities from the adverse impacts of climate change.

With respect to health outcomes, the NAPA warns that “in the Solomon Islands specific diseases have been linked to climate and or weather patterns including malaria, mental illness, malnutrition, diarrhoea, acute respiratory infections, micronutrient deficiency, parasitic diseases due to poor sanitation, tuberculosis, leprosy and non-communicable diseases” and also notes that “… such changes to health and disease place additional burden on women and children” (NAPA, 2008).

In 2010, in recognition of the need to address the knowledge gaps and improve understanding of the relationship between climate and health in the Solomon Islands, the Ministry of Health and Medical Services (MHMS) of the Solomon Islands undertook a twelve-month project, supported by the WHO South Pacific office. This research project had as one of its aims, the compilation of a National Climate Change and Health Action Plan based on the assessments undertaken and to provide recommendations to the Government on appropriate country specific adaptation responses.

The research reported here forms the second part of work carried out on the health impacts of climate change in the Pacific, the first being that for Vanuatu (Spickett et al., 2013)

### 1.2 The Assessment of the Health Impacts of Climate Change

Health Impact Assessment (HIA) was originally developed as a tool to consider potential health issues during planning stages of proposals but is now finding applications in assessing policies and programs which may affect human health. HIA uses established systematic mechanisms to identify and evaluate the factors in the proposed activity that could affect human health. HIA is commonly defined as “a combination of procedures, methods and tools by which a policy, program or project may be judged as to its potential effects on the health of a population, and the distribution of those effects within the population” (WHO, 1999). HIA aims to identify and evaluate both positive and negative impacts of activities through an evidence-based process and to provide decision makers with information about how proposed activity may affect the health of people likely to be affected (WHO, 2013).

The World Health Organisation and others (Brown et al., 2011; Nelson, 2003; WHO, 2003b) have identified that the HIA process provides an appropriate methodology by which the potential impacts of climate change on human health could be initially assessed to support decision making, especially in the consideration of the implications for health equity (Patz et al., 2008). The HIA methodology was thus used as the basis for the development and implementation of a process to identify, evaluate and prioritise the potential impacts on health of climate change and develop potential adaptation strategies for application in the Solomon Islands. The health and well-being of the community are determined by the social and economic circumstances and the environment (WHO, 2013). Further, health is dependent on the activities of a range of private and public sectors including those affecting the biophysical environment, transport, energy supply, and food supply. As these sectors can impact on health they need to be included in processes to determine the direct and indirect risks of climate change on human health and the development of potential adaptation strategies. The involvement of the public and the widest possible range of sectors in all stages of the HIA should provide those with interests the opportunity to engage in the process and act collaboratively to make the best use of possible benefits as well as to minimize potential adverse effects.

### 1.3 Climate Change in the Solomon Islands

The Solomon Islands is an archipelago of 997 islands lying just south of the equator in the Western Pacific ocean. The total land area is 28 785 square kilometres and the predominantly Melanesian population is approximately 520 000. The majority of the largely rural population depend on subsistence farming and fishing; the economy is driven mainly by agriculture, forestry, fishing, manufacturing and services. Challenges to the economic and social development of the Solomon Islands include its dispersed population, dependence on international aid and trade, and its susceptibility to natural disasters (NAPA, 2008).

The health status of Solomon Islanders is complex, as the country is among those experiencing the so-called “epidemiological transition”, with substantial impacts from both communicable diseases (including malaria and tuberculosis) and non-communicable diseases (NCDs) such as obesity, diabetes and circulatory diseases (Annual Report on the Performance of the Health Sector for Solomon Islands, 2009).

The Solomon Islands climate is tropical, with very little variation in temperature throughout the year and two seasons: wet (November to April) and dry (May to October), although the amount of rainfall varies among islands and regions.

The Pacific Climate Change Science Program (PCCSP) (2011) has identified the following five key climate change phenomena for the Solomon Islands:

Increasing air and sea-surface temperatures
  Air temperatures may rise by up to 1.0 °C by 2030 and more than 3 °C by the end of the century.
Altered rainfall patterns
  While historical trends are difficult to discern, rainfall is “generally projected to increase” over this century with more extreme rainfall events expected.
More severe cyclones
  Tropical cyclones are expected to decrease in frequency but increase in intensity.
Sea-level rise
  It is important to note that, for coastal communities and residents of low-lying islands and atolls, as little as 10-20cm of sea-level rise can cause crop failure, saline intrusion into drinking water supplies, erosion and possibly even the need for relocation.
Ocean acidification
  The increasing acidity of sea-waters in the Solomon Islands region will have a detrimental impact on coral reef ecosystems.

**The Health and Climate Change Adaptation Project**

The aims of this research were:

- To identify the potential risks of climate change to human health for the Solomon Islands communities;
- To evaluate the risks to determine their respective priorities in terms of the likelihood of the event occurring and the severity or consequences of the potential impacts on human health and safety; and
- To propose a range of feasible adaptation strategies to avoid or mitigate the most serious impacts of climate change on health in the Solomon Islands communities.

The need for a comprehensive understanding of the implications on the health and well being of the people in the Solomon Islands of climate change had been identified. The outcomes of this research provided guidance and support for the Government of the Solomon Islands through the production of an appropriate climate change and health action plan for adaptation, which included mechanisms and recommendations for its implementation.

## 2. Methods

This research project used a HIA framework and followed a sequence of three steps, which were similar to those previously reported (Spickett et al., 2013): planning the project, implementation of the project and then the development of adaptation strategies with each stage involving stakeholders from the Solomon Islands.

Health Impact Assessment is a structured process used to identify the scope of impacts to health, both positive and negative, that may arise from an activity as part of decision-making. An important focus of HIA is to address the principles of democracy, equity, sustainability and promotion of health within an evidenced based framework. Risk assessments are undertaken on each of the identified impacts and relevant management options are developed to mitigate adverse outcomes on health and well being. Within the context of climate change, HIA provides a means by which local specific environmental changes and community variables, potentially affected by climate variables, can be systematically addressed.

Health was considered in broad terms for this project and included a wide range of determinants, as given in the WHO definition of environmental health including “…*all the physical, chemical, and biological factors external to a person, and all the related factors impacting behaviours*” (WHO, 2013a).

Participants were invited to take part in the project based on their knowledge, expertise and access to data and information relevant to the Solomon Islands. The participants included Government Ministry representatives such as health, environment, meteorology and climate change, transport, agriculture, emergency and other public services, as well as private sector expert representatives. The areas represented included:

- The bio-physical environment (water, air quality, ecosystems)
- The social and economic environment (economy, mental health, communities and lifestyle, dislocation)
- The built environment and infrastructure (transport, energy, essential services)
- Environmental diseases (vectors, pests, communicable diseases)
- Food security and safety
- Disaster and management (extreme events)
- Risk assessment and management

In the process of including participants emphasis was placed on the inclusion of those with a good appreciation of the local circumstances and variability across the Solomon Islands.

The stages of the HIA of climate change for the Solomon Islands were:

**a. Planning**

An emphasis on a learning-by-doing approach for the project enabled the Ministry of Health representatives to take responsibility for implementation of the HIA for the Solomon Islands and to work closely with representatives of a range of government sectors. This qualitative approach included a series of workshops for each stage of the process held with stakeholders including considerable participation in the development of responses for adaptation.

The plan for this project generally followed the steps of the previously reported climate change and health vulnerability and adaptation assessment (Spickett et al., 2011) and used a stepwise approach (Spickett et al., 2013; Spickett et al., 2007), which enabled the participants to work through each stage in a systematic manner. The steps were divided into the phases:

Identification of the potential impacts of climate change on the health of the Solomon Island Communities;
Evaluation and prioritisation of the climate-sensitive health impacts based on the estimated risk to health each posed in the context of climate change;
Identification of potential adaptation strategies to reduce the risks posed by each identified by each climate change parameter.

**b. Implementation**

Stakeholders from sectors whose activities were considered to potentially influence the health of the community from changes to the climate were invited to take part in focus group meetings. The meetings included senior representatives from most Government sectors responsible for the development and implementation of policies and procedures. The group was provided with a scenario of potential changes in climate in the Solomon Islands for the year 2030 based on projections from PCCSP (Pacific Climate Change Science Program, 2011). Two important assumptions made for the entire project were:

1) The year is 2030 and climate change projections have occurred
2) Only the management strategies currently in place for each likely health impact are taken into account.

The group members then considered, in the context of the determinants of health (WHO, 2013), the four major areas of the physical environment, which considered impacts associated with water quality, air quality and biodiversity, the social environment, which included population displacement and mental health issues, the built environment and the impacts related to services, infrastructure and economics, including resource availability and access to a range of health, emergency and other services, as well as environmental diseases and food and the impacts related to production of food, vector-borne and food-borne disease and other environmental diseases.

The climate change effects were also divided into the broad sections of increases in severity &/or incidence of extreme events (tropical cyclones, storms, droughts and heatwaves), increases in temperature, changes in rainfall (patterns and volume), and increases in sea-level.

For each potential climate change each group then identified the potential impacts on health, health impact pathways, vulnerable groups, current responses and limitations and any gaps in knowledge. Common themes of vulnerability across a range of health impacts were considered under the categories; regional, economic, social and infrastructure and services.

In order to make some judgement about the priority areas to be addressed in the implementation of adaptation strategies a comparative measure of risk was used. Members of the stakeholder group with expertise in health or risk assessment applied a qualitative risk assessment process in their specific discipline areas to make a judgement about the potential levels of risk to public health in the Solomon Islands.

The potential impacts were divided into the following categories of health impacts:

- Extreme Events
- Temperature Increase and Related Changes
- Water-borne Disease and Water Quality
- Vector-borne diseases
- Air Quality
- Food-borne diseases
- Food Production
- Social Impact/Community Lifestyle-e.g. Dislocation, Mental Health

The health consequences and the likelihood of the health impact occurring were considered and given a risk ranking on a qualitative scale. The rankings for likelihood of the health impacts occurring considered were rare, unlikely, possible, likely and almost certain.

For consequences, the health impacts were considered in terms of the severity or magnitude of the health impact including consideration of the number of people affected, the duration of the impact and the socio-economic implications. For each overall ranking a rationale was provided. The results of this risk assessment process were entered into a risk ranking matrix and were assigned a risk ranking category of low, medium, high or extreme. The assigned risk rankings given by each group were compared and discussed. It was important that the final risk ranking level was reached by consensus to enable a focus on the high level risks and to be used as the basis for the development of potential adaptation strategies.

The risk management stage of the project considered adaptation measures for the potential impacts to health with a risk ranking of medium or higher. A list of potential adaptation measures had been identified from previous research (Spickett, 2011) and participants considered the application of each measure for the Solomon Islands. Any other measures considered appropriate were added.

**c. Adaptation Strategies**

The adaptation measures were categorised as:

- Legislative or Regulatory
- Public Education or Communication
- Surveillance and Monitoring
- Ecosystem Intervention
- Infrastructure Development
- Technological/Engineering
- Medical Intervention
- Research/Further Information

Each adaptation measure was considered in the context of its relevance for the Solomon Islands, the current capacity in the Solomon Islands to be able to address the measure including specific consideration of vulnerable groups and regions. Capacity was rated as; not in place (N); inadequate (I); being developed (D); or adequate (A). Participants were requested to consider how the adaptations could be implemented in the Solomon Islands in terms of whether an adjustment or modification of existing measures would be appropriate or whether the development of new measures was required. An important component was the identification of the sectors that would be involved in the development and implementation of each of the adaptation strategies.

## 3. Results

The application of an HIA framework addressing climate change in the Solomon Islands provided for the:

- Identification of potential health impacts
- Identification of vulnerable groups
- Understanding of key current controls or coping strategies
- Determination of current knowledge and gaps
- Identification of linkages between sectors
- Assessment of risk associated with each impact
- Identification of opportunities for adaptation and responsible sectors

The range of health problems arising from the environmental, social and economic changes that may be affected by climate change in the Solomon Islands was identified. The risk assessments relied on participants drawing on the knowledge and expertise in their particular area, however cross sector discussion and clarification was emphasised to reach consensus. Based on feedback from expert participants, which included relevant evidential information and data, climate-sensitive health risks in the Solomon Islands were ranked as per the results in Table 1.

**Table 1:** Climate-sensitive health risks in the Solomon Islands

Risk category	Health issue
Extreme	Vector-borne diseases
	Respiratory diseases
High	Water-borne diseases
	Food-borne diseases and malnutrition
	Non-communicable diseases
	Other infections and/or re-emerging diseases*
	Traumatic injuries and deaths
Medium	Temperature-related illnesses
	Circulatory disease
	Eye, ear and skin conditions
	Mental health disorders
Low	Sexually transmitted infections

* This “other” category includes mainly infectious diseases (e.g. leptospirosis, leprosy), which may be affected by climate change via various mechanisms, but for which specific adaptation strategies would be currently considered impractical. It is therefore assumed for the moment that adaptation strategies targeted at other categories (eg. water-, food- and vector-borne diseases and respiratory diseases) will, at least to some extent, cover some of the issues in the “other” category. This assumption will need to be regularly reviewed.

Consideration of vulnerable communities and regions highlighted that existing health vulnerabilities are likely to be exacerbated by climate change.

The most extreme level of risk to health from climate change was considered to be from vector-borne diseases and respiratory disease.

Potential adaptation strategies for the management of climate-sensitive health risks only for the extreme risk categories are given in Table 2. Adaptation strategies were also proposed for the health risks in the high category but they are not presented here.

**Table 2:** Potential adaptation strategies and actions for priority extreme climate-sensitive health risks in the Solomon Islands

Strategies	Actions
**EXTREME RISK – Vector-borne Diseases**	
Legislative or Regulatory	- Review regulations/guidelines for control of mosquito breeding and other vectors
	- Review building procedures to encourage the inclusion of insect-proof enclosures
	- Ensure vector (malaria) control is considered by all relevant sectors
	- Regulate and enforce the disease notification procedure
	- Provide resources at various government levels for inspection and regulation
Public Education & Communication	- Improve health promotion, disease prevention and health care of migrants and others
	- Strengthen participation of communities in health promotion activities related to vector control
	- Provide education program about the risks associated with being close to vector breeding areas
	- Prepare education materials for the needs of specific groups
	- Improve collaboration between health and other sectors
	- Build capacity building in the development and implementation of materials)
	- Include climate change and health in school curricula
	- Increase community awareness of all relevant legislation
	- Strengthen the capacity of the community through Corporate Social Responsibility programs
Surveillance & Monitoring	- Strengthen monitoring & evaluation of health promotion programs
	- Conduct surveillance of vector density and disease transmission
	- Improve capacity for monitoring and surveillance
	- Ensure surveillance programs detect vectors and diseases spread through uncontrolled movement of migrants and others
	- Strengthen laboratory capacity to support disease surveillance and prevention systems
	- Implement more effective exchange of surveillance data
	- Increase testing for exotic diseases in those coming into the country
	- Improve reporting to the National Surveillance system
Ecosystem Intervention	- Improve management of disease vector breeding sites
	- Undertake surveillance of exotic arthropod organisms with health implications
	- Improve the management of agricultural practices
Infrastructure Development	- Develop appropriate designs to minimise vector breeding potential and maintenance requirements
	- Manage vectors during periods of high risk of vector-borne disease
	- Increase collaboration between health, infrastructure, forestry, environment and conservation
	- Ensure training for designers and planners includes awareness of vector-borne diseases
	- Strengthen networks among stakeholders including government
Technology or Engineering	- Develop selective and sustainable vector controls, including preparedness for emergency control
	- Implement contingency planning to allow emergency personnel and equipment access to isolated areas
	- Improve road design so that water does not form pools
	- Modify building design to exclude rodents and other vectors.
	- Increase capacity building (training, resource allocation)
Health Intervention	- Ensure early diagnosis and treatment of all vector-borne diseases
	- Strengthen quarantine and disease response and testing in facilities
	- Train more health professionals
	- Support ongoing efforts towards development of effective vaccines (e.g. for malaria)
	- Strengthen Education of GPs and health authorities
	- Strengthen network of referral system
Research/Information	- Develop more accurate projections on climate change impacts likely to influence health impacts e.g. rainfall
	- Use cost–benefit analysis to assess the viability of intervention/monitoring programs (malaria & other)
	- Undertake quantitative risk assessments on a regional basis (malaria & other)
	- Undertake regional assessments of health issues and identification of vulnerable groups (malaria & other)
	- Undertake assessment of vector competence of native mosquitoes for exotic pathogens
	

**EXTREME RISK – Respiratory diseases**	
Legislative or Regulation	- Progress development and enforcement of air quality standards
	- Strengthen vehicle inspection procedures (for air emissions)
	- Develop indoor air quality standards (NB. highlight improved ventilation in buildings)
	- Improve waste management system (possibly the combustion of solid waste)
	- Review relevant legislation
	- Strengthen human resource capacity building to increase enforcement of legislation
	- Develop policy on stakeholder collaboration to ensure that activities are carried out effectively
	- Develop standard designs for buildings kitchen (to ensure proper ventilation)
Public Education & Communication	- Improve awareness of health effects of air pollution
	- Provide public education on the benefits to air quality by reducing car use and wood fires
	- Develop education programs for specific groups
	- Strengthen collaboration with stakeholders
Surveillance & Monitoring	- Increase monitoring of air quality (infrastructure development & monitoring system)-(indoor & outdoor)
	- Undertake regular analysis of morbidity data, hospital admissions and emergency attendance.
	- Monitor seasonal patterns of respiratory disease
	- Use ‘sentinel’ populations in different areas, in particular vulnerable groups.
	- Monitor building plans to ensure compliance with building standards (e.g. for ventilation)
Ecosystem Intervention	- Provide natural shade for UV protection.
	- Strengthen reforestation/revegetation projects to be strengthened (consider health need)
	- Implement incentives to reduce air pollutants e.g. better waste management process
Infrastructure Development	- Strengthen road management (Land transport authority)
	- Seal roads in regional and remote areas
	- Improve public transport systems to reduce reliance on cars
	- Increase shaded areas in public places
	- Increase renewable energy infrastructure
	- Improve building standards/design (e.g. to consider adequate ventilation)
	- Develop transportation facilities to reduce air pollution.
Technology or Engineering	- Reduce emissions from range of sources.
	- Implement incentives to use green power
Health Intervention	- Increase understanding of the possible links between climate change, air quality and health
	- Improve diagnosis and medical treatment for the range of potential health impacts related to air quality and UV exposure.
Research/Information	- Investigate the relationship between air pollutants and climate parameters (e.g. ozone levels may be affected by cloud cover)
	- Investigate health impacts of long-term exposure to high levels of air pollution, particularly for vulnerable groups
	- Improve understanding of potential health impacts of increased air conditioning use and increased time indoors.
	- Improve understanding of the role of aeroallergens in respiratory symptoms
	- Improve understanding of the relationship between temperature, behavioural changes and UV exposure.

## 4. Discussion

Climate change will not necessarily bring new threats to public health in the Solomon Islands, but the most likely impact is the exacerbation of the public health problems that currently exist. Society in the Solomon Islands is experiencing the so-called “epidemiological transition”, where there are relatively high burdens of both the “traditional risks” associated with infectious diseases and the “modern risks” such as non-communicable diseases. The main health impacts of climate change tend to emphasise both of these types of risks. The projected changes to rainfall patterns are expected to exacerbate current difficulties associated with the control of mosquito breeding and thus the distribution and density of disease vectors. Similarly, the increasing use of vehicles for transport and reliance on wood stoves for cooking has the potential to affect both indoor and external air quality, especially exposure to particulate matter. Identification of these as extreme risks highlighted the growing concerns among the health community of the Solomon Islands of the potential impacts to communities and the resources of the health care sector being sufficient to be able to respond to further demands on services.

The investigations for this research, identified difficulties associated with elements of uncertainty. Quantitative health risk assessment procedures use a systematic approach to characterise the nature and magnitude of risks to health and normally rely on the documentation of known risks. With the availability of good baseline data, reasonably good risk estimates can be determined. However, considering the risks to human health from climate change can be difficult due to the uncertainty about interacting climatic variations and the resulting changes to environmental parameters. Similarly, uncertainties arose about the extent and type of proposed adaptations that could be implemented, as awareness of the status of their current circumstances was limited. Further investigations of the proposed adaptation measures for reducing health impacts in the Solomon Islands were identified as an important element for future responses.

The consequence and/or likelihood levels of the potential health impacts identified during the risk assessment process also resulted in a degree of uncertainty. Levels assigned were typically higher for indirect and social health impacts, often due to the lack of knowledge about the relationships between the climate variable and the health impact and the complexity of this relationship. Of relevance was the need to consider vulnerability, particularly vulnerable groups within communities, and generally, a greater degree of conservatism was applied to the rankings.

As with other risk assessment processes there is a need to identify the level of priority for responses to the assessed risk levels. The management actions considered necessary to address climate-sensitive health issues and progress adaptation options ranged from those which require urgent attention for the extreme risks to the low risks where the current controls are considered adequate but require regular review.

Although the various sectors focused on issues specifically related to their fields of work, a range of common actions within the adaptation options for the different health impacts were consistently identified. These included the need for:

- increased capacity both in human resources and equipment and other support;
- further information on the health impacts of climate change, including incorporation of these considerations into the training curricula of health professionals in the Solomon Islands;
- community and school-based education programs starting at the primary school level, on the potential health impacts of climate change and the need for adaptation strategies;
- improvements to the collection, collation, storage and analysis of data on health status in the community;
- ongoing inter-sectoral collaboration; and
- improved standards and better enforcement of current regulations.

It was recommended that priority be given to the adaptations for the “extreme” and “high” risk categories and that a whole-of-government approach should be employed. It was identified that for each of the adaptation measures, a lead agency or sector would take responsibility for their progression but the measures would be developed in conjunction with other relevant sectors. It was recognised that the health sector should be included in all groups to identify any unexpected or unforeseen adverse health impacts.

Further investigation and analysis should determine whether each of the proposed adaptations can be implemented readily. It may be that further analysis is required to evaluate the risks and to determine the most appropriate response actions.

It should also be accepted that some risks might need to be accepted if there is no cost-effective adaptation measure that can be readily applied or that the risk is not considered significant.

The Health Impact Assessment (HIA) process used in this research project can be modified and used as a framework as new information on the monitoring of climates parameters, predicted climate changes, changes to environments and potential impacts on human health become available.

The limitations of this project have been recognised. There are many aspects of climate change impacts which may be unfamiliar to many as noted by Fussell (2008), such as the spatial scale, the long-term horizon and the complex spatial and time patterns. To allow for these factors a conservative scenario for 2030 was considered appropriate for this investigation. It was also understood that the magnitude and extent of potential health impacts from climate change should be reassessed as time progresses and climate predictions become more accurate, especially when improved regional data becomes available.

Engagement with other sectors was valuable and important. It was recognised that comprehensive consultation with all potentially affected sectors needs to be maintained.

Although the main focus of the discussions tended to be on adverse impacts, there may be opportunities for benefits to health arising from implementation of adaption responses. It is important that the potential co-benefits (for health) are also considered.

## 5. Conclusions

This process of undertaking a modified HIA approach to an assessment of climate change and health vulnerability and adaptation has provided a comprehensive and consultative approach in identifying the main climate change-attributable health impacts of concern in the Solomon Islands. The identified health impacts tend to emphasise the main public health risks that are dominant in a society in the stages of risk transition and show a mix of the traditional disease risks and some of the lifestyle risks associated within a society with excess intake of a high-energy diet and a more sedentary lifestyle.

The possible environmental changes, which could result in impacts on health, the estimated levels of risk and the estimated current level of capacity response, were considered for the Solomon Islands. Consequently, an extensive range of potential adaptation strategies that could reduce or mitigate the adverse impacts of climate change on human health and that may include some potential benefits to health was identified. Based on these assessments, relevant sectors should be better positioned to make judgements about the risks to health associated with their activities and implement appropriate responses that require attention in the short term, as well as consider those that could be dealt with at a later stage or those where more information is required. Decision makers can use the results to provide direction on planning for the short, medium and long term. With a more comprehensive understanding of the predicted climate variability to be experienced by the Solomon Islands, the procedure used can be modified to further develop more extensive and responsive adaptation strategies for protection and promotion of health and well-being.

In the final analysis there may be some risks that will need to be accepted because there are limited cost-effective adaptation measures available or insufficient evidence of the risks to human health. An indication of the types of adaptation responses needed to reduce the adverse effects of climate change on health has been provided, however, further information is required to enable a more detailed assessment of current and potential adaptation measures. Specific sectors will need to increase their general awareness and capacity to enable the impacts of climate change on health to be more accurately predicted. This will result in further effective adaptation strategies.

Improvements in environmental and health monitoring and surveillance systems by the health care sector across the Solomon Islands will be required including the establishment of monitoring programs for specific vulnerable groups to provide sentinel data. The potential barriers to the introduction of health adaptation strategies and their cost effectiveness require further investigation and research and should be linked with climate change activities of other organisations.

It is accepted that climatic conditions in the Solomon Islands are changing and that physical and environmental changes will influence the lives of Solomon Islanders. This project has identified a number of potential health impacts that may arise from climate change in the Solomon Islands, and has considered mechanisms by which these could be managed. Some of the more serious impacts of climate change on health have been identified and potential adaptation responses that could be implemented across the Solomon Islands identified. The HIA process provides a mechanism by which potential health impacts from climate change can be identified and assessed and enables the development of adaptation responses. The development of a national Climate Change and Health Action Plan using this process has resulted in a comprehensive strategy with clear identification of the risks to health for use by the Government to protect and enhance the health of the Solomon Islands community into the future.
